# Accurate Detection and Quantification of the Fish Viral Hemorrhagic Septicemia virus (VHSv) with a Two-Color Fluorometric Real-Time PCR Assay

**DOI:** 10.1371/journal.pone.0071851

**Published:** 2013-08-20

**Authors:** Lindsey R. Pierce, James C. Willey, Vrushalee V. Palsule, Jiyoun Yeo, Brian S. Shepherd, Erin L. Crawford, Carol A. Stepien

**Affiliations:** 1 Great Lakes Genetics/Genomics Laboratory, Lake Erie Center and Department of Environmental Sciences, The University of Toledo, Toledo, Ohio, United States of America; 2 Department of Medicine, The University of Toledo, Toledo, Ohio, United States of America; 3 ARS/USDA/University of Wisconsin at Milwaukee/School of Freshwater Sciences, Milwaukee, Wisconsin, United States of America; University of Kansas Medical Center, United States of America

## Abstract

Viral Hemorrhagic Septicemia virus (VHSv) is one of the world's most serious fish pathogens, infecting >80 marine, freshwater, and estuarine fish species from Eurasia and North America. A novel and especially virulent strain – IVb – appeared in the Great Lakes in 2003, has killed many game fish species in a series of outbreaks in subsequent years, and shut down interstate transport of baitfish. Cell culture is the diagnostic method approved by the USDA-APHIS, which takes a month or longer, lacks sensitivity, and does not quantify the amount of virus. We thus present a novel, easy, rapid, and highly sensitive real-time quantitative reverse transcription PCR (qRT-PCR) assay that incorporates synthetic competitive template internal standards for quality control to circumvent false negative results. Results demonstrate high signal-to-analyte response (slope = 1.00±0.02) and a linear dynamic range that spans seven orders of magnitude (R^2^ = 0.99), ranging from 6 to 6,000,000 molecules. Infected fishes are found to harbor levels of virus that range to 1,200,000 VHSv molecules/10^6^ *actb1* molecules with 1,000 being a rough cut-off for clinical signs of disease. This new assay is rapid, inexpensive, and has significantly greater accuracy than other published qRT-PCR tests and traditional cell culture diagnostics.

## Introduction

Molecular diagnostic tools have facilitated the early detection, prevention, and spread of many important pathogens [1], led by the speed, sensitivity, and accuracy of Polymerase Chain Reaction (PCR)-based assays [2]. Their ability to diagnose targeted genetic sequences and quantify levels of infectious agents with hybridization probes has advanced screening technology for multiple human diseases, including influenza, hepatitis, and HIV [3], [4]. Use of these approaches to elucidate and characterize plant and animal pathogens likewise is growing at a rapid pace [5], [6].

Viral Hemorrhagic Septicemia virus (VHSv) causes one of the world's most serious finfish diseases, infecting >80 species across the Northern Hemisphere [7], yet there remains a need for a fast, sensitive, accurate, and inexpensive diagnostic test. VHSv is a negative-sense, single stranded RNA *Novirhabdovirus* of ∼ 12,000 nucleotides, with six open reading frames of *3′N-P-M-G-Nv-L*’5 [8]. Infected fishes often swim erratically and have bulging eyes, distended abdomens, and extensive external/internal hemorrhaging [9]. The virus survives for up to 13 days in the water [10], and can be spread via ballast water, boating, equipment, and aquatic animals (e.g. birds, turtles, leeches, and amphipod crustaceans) [11]–[14]. It is transmitted most readily during the spring spawning season through fish waste, reproductive fluids, and skin secretions [11].

VHSv first was described in the late 1930s as “*Nierenschwellung*” in aquacultured rainbow trout (*Oncorhynchus mykiss*) from Europe [15]. It now occurs across the Northern Hemisphere as four genetically and geographically distinct strains (I–IV) and substrains, whose evolutionary and biogeographic patterns recently were analyzed by Pierce and Stepien [16]. Strains I–III primarily occur in Europe, where they infect a wide variety of marine, estuarine, and freshwater fishes. Strain IV (now classified as IVa; [17]) first was discovered in 1988 from North American Pacific coastal fishes, including salmonids [18], [19], and now also occurs in Japan [20]. In 2000, another IV substrain (now designated as IVc per [16]) was discovered off the coast of New Brunswick, Canada, infecting the estuarine mummichog (*Fundulus heteroclitus*) and three-spined stickleback (*Gasterosteus aculeatus*) [21]. In 2003, a new and especially virulent substrain, IVb, was described from a moribund muskellunge (*Esox masquinongy*) in Lake St. Clair of the freshwater Laurentian Great Lakes system [17]. Substrain IVb since has spread throughout all five of the Great Lakes, infecting >30 species, including many commercially and ecologically important fishes, such as muskellunge, drum (*Aplodinotus grunniens*), walleye (*Sander vitreus*), yellow perch (*Perca flavescens*), and round goby (*Neogobius melanostomus*). Substrain IVb now contains at least 16 glycoprotein (*G*)-gene sequence variants [22], whose rapid spread and diversification in a quasispecies mode suggest that this strain mutates rapidly and may be highly adaptable (see [16]).

To avoid outbreaks of the virus, the Aquatic Invasive Species Action Plan [23] requires that aquaculture and baitfish vendors from U.S. states (Illinois, Indiana, Michigan, Minnesota, New York, Ohio, Pennsylvania, and Wisconsin) and Canadian provinces (Ontario and Quebec) have their fish products certified as VHSv-free prior to interstate or international transport. Cell culture is the VHSv diagnostic that is approved by the World Organization for Animal Health [24], along with the joint Fish Health Section of the U.S. Fish and Wildlife Service and the American Fisheries Society [25]. The cell culture process takes a month or longer for cell growth, cell confluency, viral growth, and confirmation PCR. It moreover lacks the sensitivity to detect low viral concentrations in carrier fish, and results in false negative levels reported as 43–95% [26]–[28].

Real-time quantitative reverse transcription (qRT)-PCR assays for detecting VHSv [26]–[34] likewise have substantially high false negative rates that ranged from 15–92% [26]–[28]. The high false negative rates in those assays may have resulted from unknown and/or unmonitored effects from interfering substances in the PCR or reverse transcription reactions (rxn), which circumvented detection of the target gene [35].

**Figure 1:**
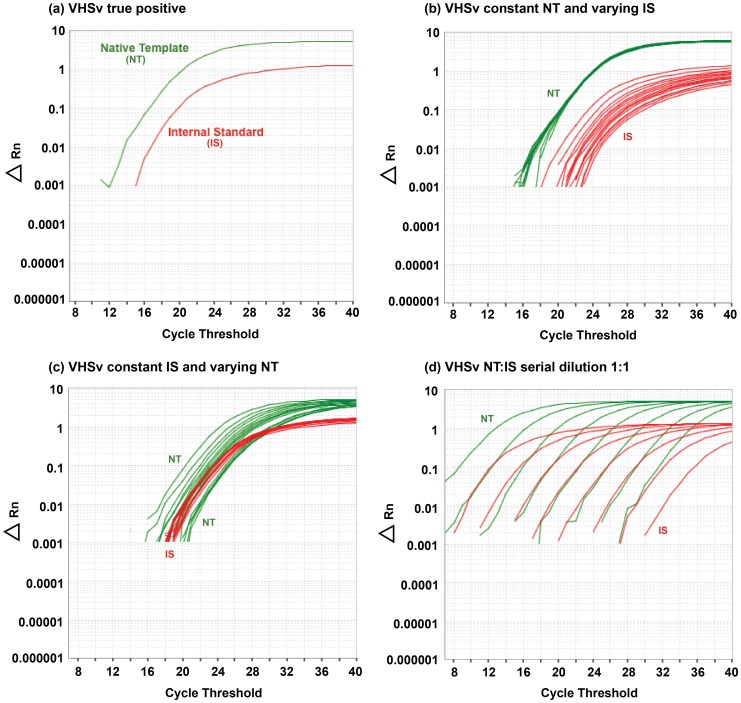
Real-time PCR amplification plots for various experiments. ABI 7500 real-time PCR results for (a) a true VHSv positive fish sample, (b) the relationship between the VHSv Native Template (NT) and Internal Standard (IS) with the NT held constant and the IS varied, (c) the relationship between VHSv NT and IS with the IS held constant and the NT varied, and (d) the relationship between VHSv NT:IS with concentrations held constant for dilutions of 1∶1–1∶20. Green = NT, red = IS, y = fluorescence of the reporter dye minus the baseline (Δ fluorescence), x = cycle threshold.

To avoid those issues, the present research describes and evaluates a new, accurate, fast, and highly reliable assay to diagnose and quantify VHSv. This assay uses Standardized Reverse-Transcriptase Polymerase Chain Reaction, i.e. StaRT-PCR, which is a form of competitive template RT-PCR that yields rapid, reproducible, standardized, and quantitative measurement of data for many genes simultaneously [36]. StaRT-PCR uniquely incorporates internal standards (IS) in the rxn mixture to improve accuracy and circumvent false negative results. Our new assay is based on real-time PCR equipment that is readily available in most diagnostic laboratories, markedly improving on a previous version of our VHSv test [37], which also used StaRT-PCR, but relied on less common capillary electrophoresis equipment (i.e. Agilent; Agilent Technologies, Santa Clara, CA). In the present study, results from both assays are evaluated by us to determine the presence or absence of VHSv and measure concentration of the virus from fish samples in targeted field and laboratory studies. We assay the VHSv nucleoprotein (*N*)-gene and the fish reference beta-actin 1 (*actb1*) gene, assessing amplification relative to known numbers of their respective competitive IS molecules. Our new approach uses sequence specific fluor-labeled hydrolysis probes that can be used on a variety of real-time PCR thermal cyclers, on which positive VHSv results are visible as two colors on the real-time PCR plot (see Figure 1a; green = native template (NT), red = IS).

Results of this 2-color fluorometric assay are compared to those from our previously reported Agilent capillary electrophoresis test [37], SYBR® green qRT-PCR, and cell culture [24], using the same biological samples. The numbers of VHSv molecules are quantified from field-caught and laboratory-challenged VHSv-infected fish samples with the new 2-color fluorometric assay in comparison to the Agilent capillary electrophoresis test [37].

## Materials and Methods

### VHSv Assay Development

All primers and NT probes were matched to homologous sequences of the VHSv *N*-gene, based on all VHSv strains and substrain variants from NIH GenBank (http://www.ncbi.nlm.nih.gov/genbank/) and the literature, using Biosearch Technologies Real Time Design software (Novato, CA; http://www.biosearchtech.com/). The original muskellunge isolate MI03GL from the Great Lakes (GenBank Accession no. DQ427105) served as the reference for VHSv, and *actb1* mRNA from the yellow perch *Perca flavescens* (AY332493) was used as the fish reference gene sequence. Selection criteria included: product lengths that were <100 bp, with optimal melting temperatures of 65–68°C for primers and 68–72°C for probes. NT probes for the target and reference genes were labeled with FAM (fluorescein amidite dye).

The competitive template IS probes for the VHSv *N*-gene and the fish reference *actb1* gene each were constructed by altering 5–6 bp of the NT probe sequences, and were labeled with Quasar dye having 670 nm maximum absorbance (Biosearch Technologies). The IS probes were designed to: minimize cytosine and thymine (30%), maximize adenine (50%), avoid guanine at the 5′ end, have lengths <24 bp, and have predicted melting temperatures ±0.02°C of the NT probe. Synthetic NT and IS templates for VHSv and *actb1* were assembled by combining the forward primer, probe, and connecting sequence through the reverse primer (Table 1), and were synthesized by Life Technologies (Grant Island, NY; http://www.lifetechnologies.com/us/en/home.html). The BLAST procedure (http://blast.ncbi.nlm.nih.gov/Blast.cgi) was employed to verify that all primers, probes, and IS did not recognize other viral or fish DNA sequences.

**Table 1:** Sequences and PCR parameters for our 2-color fluorometric VHSv assay.

Primers, Probes, and IS	Nucleotide position		Sequence (5′-3′)	*T*_m_ (°C)	GC%	Product length (bp)
*N*-gene:						
pVHS4b F2	316–336		GCC GGA ATC CTT ATG CCG ATG	68.0	57.1	74
pVHS4b R2	367–389		CCC TTG ACG ATG TCC ATG AGG TT	67.0	52.0	74
Housekeeping Gene:						
p*actb1* F4	1075–1095		CCC ACC AGA GCG TAA ATA CTC	65.5	52.0	92
p*actb1* R4	1145–1165		CTC CTG CTT GCT GAT CCA CAT	65.6	52.0	92
Probes:						
pVHS 4b 1NT	342–359	ACT GGC CCA GAC TGT CAA		68.5	55.6	18
VHS4b IS	342–358	*TG* T GGC C*G*A G*T*C *A*GT C*C*		70.3	64.7	17
p*actb1*_4 NT	1098–1116	TCT GGA TCG GAG GCT CCA T		71.8	57.9	19
p*actb1*_4 IS	1098–1117	*A* CT *C*GA T*T*G GAG G*G*T CC*G AC*		71.8	60.0	20
Synthetic Templates:						
VHS 4b NT	304–416	ACT GGC ATC GAG GCC GGA ATC CTT ATG CCG ATG AAG GAA CTG GCC CAG ACT GTC AAC GCC GAC AAC CTC ATG GAC ATC GTC AAG GGG GCC CTG ATG ACG TGT TCC CTT CTG AC		82.2	56.6	113
VHS4b IS2	304–416	ACT GGC ATC GAG GCC GGA ATC CTT ATG CCG ATG AAG GA*T G*TG GCC *G*AG *T*C*A* GTC *C*AC GCC GAC AAC CTC ATG GAC ATC GTC AAG GGG GCC CTG ATG ACG TGT TCC CTT TCG AC		82.6	58.4	113
p*actb1*_4 NT	1075–1095	CCC ACC AGA GCG TAA ATA CTC TGT CTG GAT CGG AGG CTC CAT CCT GGC CTC TCT GTC CAC CTT CCA GCA GAT GTG GAT CAG CAA GCA GGA G		85.3	57.1	91
p*actb1*_4 IS	1145–1165	CCC ACC AGA GCG TAA ATA CTC TG*A* CT*C* GAT *T*GG AGG *G*TC C*GA C*CC TGG CCT CTC TGT CCA CCT TCC AGC AGA TGT GGA TCA GCA AGC AGG AG		85.5	57.6	92

Primers, probes, internal standards (IS), and synthetic templates are specified. F = forward primer, R = reverse primer, NT = native template. *Italics* = modified nucleotides in NT probe.

To ensure that the probes did not bind to non-homologous template, their specificities were tested using synthetic templates for the VHSv *N*- and *actb1* genes. Both synthetic templates (NT and IS) were serially diluted 10-fold from 10^−11^ M to 10^−15^ M and tested with all probes in PCR amplification experiments, following the directions for “Performing the VHSv Assay”, as detailed below. For example, the VHS *N*-gene IS synthetic template was evaluated with the VHS *N*-gene IS probe, as well as the VHS *N*-gene NT probe, and vice versa. The same was done for *actb1.* Cycle thresholds (C_t_) from the homologous and non-homologous templates were compared at each dilution, and the non-homologous amplifications were calculated with formula 2^(−ΔCt)^ and multiplied by the known number of input copies. If the resulting numbers of molecules were >10% of the known input copy number, then the probe was re-designed, and the process was repeated.

After synthesis, the NT and IS for each gene were PCR amplified (Table 1) in 10 individual 10 µl rxns, containing 1 µl 750 nM of each primer, 0.5 U Go-TAQ polymerase (Promega, Madison, WI), 1 µl 10X MgCl_2_ PCR buffer, 0.2 mM dNTPs, and RNAse-free water on a Rapid Cycler 2 (Idaho Technology, Inc., Salt Lake City, UT; www.biofiredx.com/). Rxns were run for 35 cycles of 5 sec at 94°C, 10 sec at 58°C, and 15 sec at 72°C, with a slope of 9.9. To purify the NT and IS, all 10 replicate PCR products per template were combined into a single tube, loaded onto individual 2% low melting pre-cast agarose gels from E-Gel iBase (Invitrogen, Grand Island, NY; www.invitrogen.com/), separated by electrophoresis, and visualized on a UV transilluminator. The NT and IS bands for the VHSv *N*- and *actb1* genes were harvested from their respective gel collection wells. Mean concentrations (ng/µl) of each were calculated from 1 µl of their purified products as measured with an Agilent 2100 Bioanalyzer in triplicate, and converted into molarities according to the formula (1):



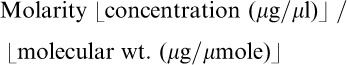
(1)


To control for inter-sample and inter-experimental pipetting variation, a synthetic internal standard mixture (ISM) was created with the purified IS described above. To prepare the original stock ISM “A”, we estimated the relative concentrations of the VHSv *N*-gene and *actb1* IS needed to achieve a 1∶1 cDNA NT:IS relationship in a variety of samples (Table 2). Briefly, we mixed 10^−10^ M of the VHSv *N*-gene IS and 10^−11^ M of the *actb1* IS in an initial stock, labeled ISM “A”. To measure various levels of gene expression, other ISM mixtures (ISM B–H) were constructed using 10-fold serial dilutions of the VHSv *N*-gene relative to a constant concentration of the *actb1* gene IS at 10^−11^ M (Table 2). Additional 10-fold dilutions of each ISM (A–H) stock then were made with 0.1 ng/µl yeast tRNA carrier (Invitrogen, Carlsbad, CA) to prevent adherence of negatively charged IS molecules to the tube or pipette tip surfaces (Table 2, rows 2–8).

**Table 2:** Concentrations for the 2-color fluorometric VHSv assay.

A	B	C	D	E	F	G	H
−11/−10	−11/−11	−11/−12	−11/−13	−11/−14	−11/−15	−11/−16	−11/−17
−12/−11	−12/−12	−12/−13	−12/−14	−12/−15	−12/−16	−12/−17	
−13/−12	−13/−13	−13/−14	−13/−15	−13/−16	−13/−17		
−14/−13	−14/−14	−14/−15	−14/−16	−14/−17			
−15/−14	−15/−15	−15/−16	−15/−17				
−16/−15	−16/−16	−16/−17					
−17/−16	−17/−17						

Dilution mixtures (A–H) used for the Internal Standards Mixture (ISM) *actb1*/VHSv are given in units of 10^x^ M.

An External Standardized Mixture – ESM (comprised of the synthesized NT and IS for the VHSv *N*- and *actb1* genes) – was made to control for inter-lot and inter-experimental variation in probe fluorescence intensity, guard against inter-experimental variation in C_t_ selection, and normalize the probe (see equation (2), “Correction for variation in fluorescence among probes”). Stock ESM containing 10^−11^ M NT/10^−11^ M IS for the VHSv *N*- and *actb1* genes was diluted to a working concentration of 10^−13^ M NT/10^−13^ M IS and 10^−14^ M NT/10^−14^ M IS with yeast tRNA (Invitrogen).

### Fish Samples used to Evaluate the VHSv Assay

Spleen tissues from a variety of fish samples were used to test our assay for the presence and concentration of VHSv (and to compare our results to other assays, using the same samples). Fish were obtained, maintained, anesthetized, and sacrificed following the Institutional Animal Care and Use Committee (IACUC) approved protocols from the University of Toledo (#106419), Michigan State University (MSU; East Lansing, MI) (#AUF 07/07-123-00), and the U.S. Geological Survey's (USGS) Western Fisheries Research Center Challenge Facility (WFCCF; Seattle, WA) (#2008-17). Fish were euthanized with an overdose of 25 mg/ml tricaine methanesulfonate (MS-222; Argent Chemical Lab, Redmond, WA) and decapitated to ensure death. To remove any external viral particles, each fish was washed separately by submerging it 3X in double distilled H_2_O. The surgical site (anus to operculum) was disinfected with 100% ethanol and betadine using sterile equipment. Spleen tissue was removed, placed into individual 1.5 mL eppendorf tubes, flash frozen in liquid nitrogen or stored in RNAlater (Qiagen), and kept at −80°C until further processing. Gloves and all equipment were changed between each fish to ensure sterile conditions. Specimens were disposed of following the respective approved biohazard protocols of the University of Toledo, MSU, and USGS.

Samples tested for VHSv included cDNA from 23 wild-caught Great Lakes fishes, including 10 infected and 13 negatives: two bluegill (*Lepomis macrochirus*), a brown bullhead (*Ameiurus nebulosus*), a freshwater drum, seven largemouth bass (*Micropterus salmoides*), a smallmouth bass (*Micropterus dolomieu*), and 11 lake herring (*Coregonus artedi*). We also tested 40 fish from VHSv laboratory challenge experiments, including 20 muskellunge (15 VHSv infected and 5 negative controls) from the MSU-Aquatic Animal Health Laboratory (AAHL), and 20 yellow perch (14 VHSv-infected and 6 negative controls) from USGS-WFRCCF.

A series of laboratory challenge experiments were conducted by MSU-AAHL on certified VHSv-free juvenile muskellunge (Rathburn National Fish Hatchery, Moravia, Iowa) under MSU IACUC protocols AUF 07/07-123-00. Muskellunge were challenged via water immersion for 90 min with VHSv-IVb (isolate MI03GL) at 4.0×10^3^ pfu/ml, and the negative controls with 1 ml sterile maintenance minimum essential media. Fish then were placed into clean VHSv-free water, and later randomly sacrificed at pre-determined intervals, as previously described.

We also analyzed RNA from a series of juvenile yellow perch laboratory challenge experiments, using six-month-old (VHSv-certified-free) Choptank broodstrain [42] from the University of Wisconsin-Milwaukee’s Great Lakes WATER Institute (Milwaukee, WI), which were conducted at USGS- WFRCCF under their 2008–17 IACUC protocol. Perch were challenged either via intra-peritoneal injection of 1.0×10^5^ pfu/ml VHSv-IVb (strain MI03GL) or with immersion for two hours in the same dosage, while control groups had a dose of minimum essential media. Fish were selected randomly in days 0–6 for euthanization with 240 mg/L MS-222 and 1.2g/L NaHCO_3_. Dissection followed protocols described above.

### Performing the VHSv Assay

Spleen tissue (0.25–0.50 g) was ground using a sterile mortar and pestle under liquid nitrogen, and its RNA was extracted with the TriREAGENT® (Molecular Research Center, Inc., Cincinnati, OH) protocol. The RNA was re-suspended in 30 µl RNase-free water, quantified with a NanoDrop 2000 Spectrophotometer (Thermo Fisher Scientific, Waltham, MA), and adjusted to a 1 µg RNA/µl concentration. DNA-*free* DNase Treatment and Removal Reagents (Ambion Life Technologies, Grand Island, NY) were used to eliminate any contaminating gDNA. The purified RNA was reverse-transcribed to cDNA with 1 µg RNA, 5X First Strand buffer, 10 mM dNTPs, 0.05 mM random hexamers, 25 U/µl RNasin, and 200 U/µl M-MLV in a 90 µl rxn volume at 94°C for 5 min, 37°C for 1 h, and 94°C for 5 min. The cDNA was stored at –20°C.

A set of PCR rxns was run per each cDNA sample to determine the appropriate concentrations of NT and IS for *actb1* to achieve a ratio of >1∶10 and <10∶1 of amplified products. Once the NT:IS products were in balance, the VHSv *N*- and *actb1* target genes were pre-amplified simultaneously to increase the signal (i.e. lower C_t_) relative to non-specific background.

For each pre-amplification, a 10 µl volume of a master mixture was prepared with 5 µl TaqMan® Universal Master Mix II (without uracil N-glycosylase; Applied Biosystems International (ABI), Grand Island, NY), 1 µl of 10X primer solution (final concentration: 75 nM) of the forward and reverse primers for the VHS *N* and *actb1* genes (mixed together), and RNAse-free water. Eight µl of this master mixture was dispensed into individual wells of 0.1 mL 96-well TempPlate® (USA Scientific, Inc.; www.usascientific.com/) containing 1 µl cDNA and 1 µl of the appropriate ISM concentration (Table 2). This was done in triplicate to allow calculation of the mean and standard error (S.E.) of the relative VHSv *N*-gene concentration/10^6^ *actb1* molecules per fish sample. The plates then were sealed with a TempPlate® RT Optical Film and centrifuged for 2 min at 2000 rpm. PCR rxns were conducted on an ABI 7500 Fast using standard mode cycling conditions: 10 min at 95°C, followed by 13 cycles of 15 sec at 95°C and 1 min at 60°C. For the Poisson distribution experiments, 25 pre-amplification cycles were used due to lower amount of starting template. Three no-template controls per rxn, located on separated areas on the plate, were used to control for possible contamination.

A second round of amplification was performed, in which each pre-amplified sample was diluted 1000-fold with TE buffer (10 mM Tris-Cl, 0.1 mM EDTA, pH 7.4); 2 µl of each diluted product was placed into each well of a new 0.1 mL 96-well TempPlate®, along with 18 µl of a master mixture containing 10 µl TaqMan® Universal Master Mix II (without uracil N-glycosylase), 2 µl of each 10X primer solution (final concentration: 750 nM), 2 µl of each NT and IS probe (final concentration: 200 nM), and RNAse-free water. This second amplification was conducted as described above, except run for 40 cycles. The number of VHSv molecules/10^6^ *actb1* molecules was calculated using equations (2) and (3) below.



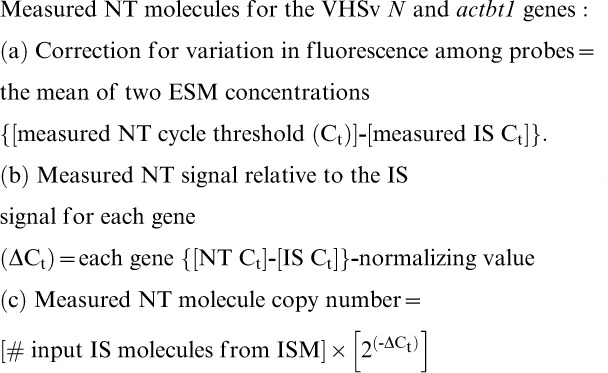
(2)




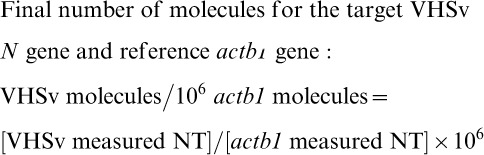
(3)


### Specificity, True Accuracy, and Linearity

Our assay was tested for non-specific amplification using two human viruses (Encephalomyocarditis virus and Vesicular Stomatitis virus) and five fish viruses related to VHSv (Hirame rhabdovirus, Infectious Hematopoietic Necrosis virus, Infectious Pancreatic Necrosis virus, Spring Viremia of Carp virus, and Snakehead rhabdovirus). The Snakehead rhabdovirus is the nearest relative to VHSv, with 62% sequence similarity [8], [16]. Twenty-five VHSv isolates were tested to evaluate amplification across a range of European, Asian, and North American variants (Table 3), encompassing all four strains. All samples were assayed in triplicate.

**Table 3:** Specificity of the 2-color fluorometric test.

Type	Isolate	Result
Human:		
Encephalomyocarditis virus		–
Vesicular Stomatitis virus		–
Fish:		
Hirame rhabdovirus^a^		–
Infectious Hematopoietic Necrosis virus (strain 220-90)^a^		–
Infectious Pancreatic Necrosis virus		–
Snakehead rhabdovirus^a^		–
Spring Viremia of Carp virus^a^		–
VHSv:		
I	DK-F1^b^	+
Ia	FR0771^b^	+
Ia	JP96KRRV9601^b^	+
II	FI-ka663-06^c^	+
III	GH35^d^	+
III	GH 44^d^	+
III	SC2645^d^	+
III	SM2897^d^	+
IVa	Bogachiel^b^	+
IVa	Cod’91^b^	+
IVa	Elliott Bay^b^	+
IVa	JP96Obama^b^	+
IVa	Makah^b^	+
IVa	Orcas^b^	+
IVb	MI03GLa,b	+
IVb	vcG002^a^	+
IVb	vcG003^a^	+
IVb	vcG004^a^	+
IVb	vcG005^a^	+
IVb	vcG006^a^	+
IVb	vcG007^a^	+
IVb	vcG008^a^	+
IVb	vcG009^a^	+
IVb	vcG010^a^	+
IVc	CA-NB00-02^e^	+

– = negative result (no amplification), + = positive result.

Isolates obtained from:

a Western Fisheries Research Center, USGS, Seattle, WA, USA.

b Cornell University College of Veterinary Medicine, Ithaca, NY, USA.

c Finnish Food Safety Authority Evira, Finland.

d Universidad de Santiago de Compostela, Spain.

e Fisheries and Oceans Canada, Pacific Biological Station, BC, Canada.

To measure true accuracy – the agreement between a measurement and its known value [38] – the relationship between the observed versus expected numbers of VHSv *N*-gene and *actb1* molecules based on Poisson analysis was determined [39]. Ten replicates were amplified for the VHSv *N*- and *actb1* genes over a series of limiting PCR dilutions, which were predicted to contain 40, 20, 10, 7, 6, 5, 4, 2, 1, 0.7, 0.4, and 0.1 molecules. Linear regression analysis was performed in the R statistical software suite v2.15.2 [40]. A χ^2^ test (in Microsoft Excel) compared the number of molecules measured with the 2-color fluorometric assay versus those from the Agilent 2100 Bioanalyzer, at the same dilutions.

Linearity was measured over two series of dilution experiments to: 1) determine the maximum and minimum ratio of NT to IS that produced reproducible results, and 2) verify that our test followed a linear trend in calculating the expected number of molecules per dilution. The first dilution set was made by mixing a constant amount of synthetic NT with decreasing amounts of IS to generate dilutions of: 1∶1 (6×10^4^ molecules), 1∶2, 1∶3, 1∶4, 1∶5, 1∶6, 1∶7, 1∶8, 1∶9, 1∶10, 1∶12, 1∶14, 1∶16, 1∶18, and 1∶20 (3×10^3^ molecules) for both genes. Identical procedures were performed by holding the IS constant, while varying the NT. The second dilution series evaluated linearity for the VHSv *N*- and *actb1* genes using 10-fold serial dilutions of the ESM at a 1∶1 ratio, with dilutions of 6×10^6^, 6×10^5^, 6×10^4^, 6×10^3^, 6×10^2^, 60, 6, and 0.6 molecules. Regression analyses were conducted to determine correlation (*R* ^2^), slope (linearity), and relation to a linear trend (*F*) among the various dilutions of NT:IS and IS:NT for each gene. Imprecision was reported as the coefficient of variation (CV), calculated as the standard deviation divided by the mean of triplicate measurements at each dilution (reported in %) (in Microsoft Excel) [41]. In addition, S.E. was calculated for each sample. For these linearity experiments, PCR was done as specified above in “Performing the VHSv Assay”, but substituting the cDNA and ISM with either 2 µl of the ESM (from dilution 1) or a concentration of 1∶1 NT/IS (dilution 2). Each dilution was run in triplicate, with a negative/no template control for each run.

### VHSv Detection Comparisons of Our Assay to Others

Results from the new 2-color fluorometric test are compared to those from our prior Agilent capillary electrophoresis assay [37], conventional SYBR® green qRT-PCR, and cell culture to evaluate their relative abilities to detect VHSv in 63 fish samples (see “Fish Samples used to Evaluate the VHSv Assay”). All samples were analyzed in triplicate and all runs had positive and negative controls. Each PCR rxn included a known cell culture positive, a negative VHSv cDNA, and a reagent negative control (nuclease-free H_2_O). PCR products were visualized on 1% agarose gels to confirm positive/negative results. The amount of yellow perch fish tissue available from the USGS laboratory challenge experiments precluded analysis with cell culture. χ^2^ tests [43] were used to compare the results among the approaches.

SYBR® green qRT-PCR experiments used a Mastercycler Realplex Thermocycler (Eppendorf, Inc., Westbury, NY) in 25 µl rxns, containing 0.05 µg of each primer (the same primers used for the Agilent capillary electrophoresis assay [37]), 2 µl cDNA product, 10 µl SsoFast SYBR® green mix, and RNase-free water. Amplifications were run on a Mastercycler Realplex Thermocycler (Eppendorf, Inc., Westbury, NY), with initial denaturation of 5 min at 95°C, followed by 40 cycles of 30 sec at 95°C, and 1 min at 60°C.

Cell culture was performed at MSU’s AAHL by M. Faisal and R. Kim, following standard OIE [24] procedures. If results were positive, RNA was extracted from infected cells as described above, reverse transcribed with Affinity Script Multiple Temperature Reverse Transcriptase PCR (Stratagene, La Jolla, CA), and amplified following OIE [24].

### VHSv Quantification using Our Assay

Positive samples were quantified with our new 2-color fluorometric real-time PCR assay and compared to our earlier results from the Agilent capillary electrophoresis procedure [36] for the 63 test fish, with linear regression in R and an *F*-test [43]. Numbers of VHSv/10^6^ *actb1* molecules were measured in triplicate, from which means and S.E. were calculated. Relative numbers of VHSv molecules were compared between laboratory challenged muskellunge showing clinical signs of infection (e.g. external hemorrhages; *N = *9) versus those without signs (*N = *9). A χ^2^ test (Microsoft Excel) was used to determine if a threshold number of VHSv molecules characterized the appearance of the clinical signs. Due to limited sample size, a power analysis (G*Power2; [44]) was used to estimate the number of fish needed to achieve 95% confidence, with an effect size of 0.50 [45].

## Results

### Performance of Our 2-Color Fluorometric Assay for VHSv

Our test results are negative for all other viruses (i.e. did not result in amplification; Table 3), including human viruses (Encephalomyocarditis virus and Vesicular Stomatitis virus) and fish viruses that are related to VHSv (i.e. Hirame rhabdovirus, Infectious Hematopoietic Necrosis virus, Infectious Pancreatic Necrosis virus, Spring Viremia of Carp virus, and Snakehead rhabdovirus). All four VHSv strains (I–IV) and all substrains evaluated (I, Ia, II, III, IVa, IVb, and IVc) yield positive amplification results with our test (Figure 1a; Table 3). Thus this new assay is specific for VHSv.

**Figure 2:**
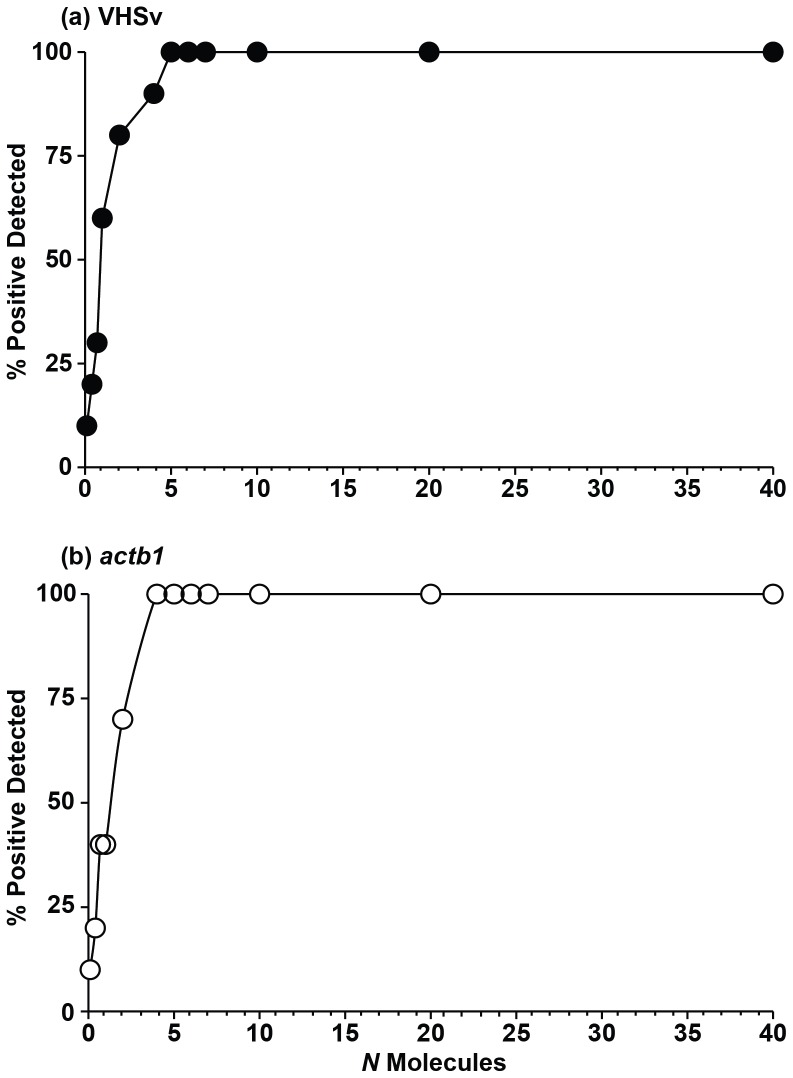
True accuracy of the 2-color fluorometric test. Results are based on % positives from 10 separate runs of 12 dilutions using a known Internal Standard Mixture (ISM). Dilutions are: 40, 20, 10, 7, 6, 5, 4, 2, 1, 0.7, 0.4, and 0.1 molecules. The 2-color fluorometric test yields 100% positives for (a) ≥ 5 molecules of VHSv and (b) ≥ 4 molecules for *actb1*.

Amplification results for the VHSv *N*-gene (Figure 2a) are 100% (10/10 times) for dilutions of 5–40 VHSv molecules, 90% (9/10 times) for 4 molecules, 80% (8/10) for 2 molecules, 60% (6/10) for a single molecule, 30% (3/10) for 0.7 molecules, 20% (2/10) for 0.4 molecules, and 10% (1/10) for 0.1 molecules (*R* ^2^ = 0.98, *F = *541.50, df = 1, 10, *p*<0.001). Values for amplification of the fish *actb1* gene are similar (Figure 2b), yielding 100% positives for 4–40 molecules (10/10), 70% at 2 molecules (7/10), 40% for a single molecule and for 0.7 molecules (4/10), 20% for 0.4 molecules (2/10), and 10% at 0.2 molecules (1/10) (*R* ^2^ = 0.97, *F = *283.60, df = 1, 10, *p*<0.001). Results indicate that the numbers of ISM molecules measured by our assay match those from the bioanalyzer for the VHSv *N*-gene (χ^2^ = 0.18, df = 11, NS) and the *actb1* gene (χ^2^ = 0.23, df = 11, NS).

**Figure 3:**
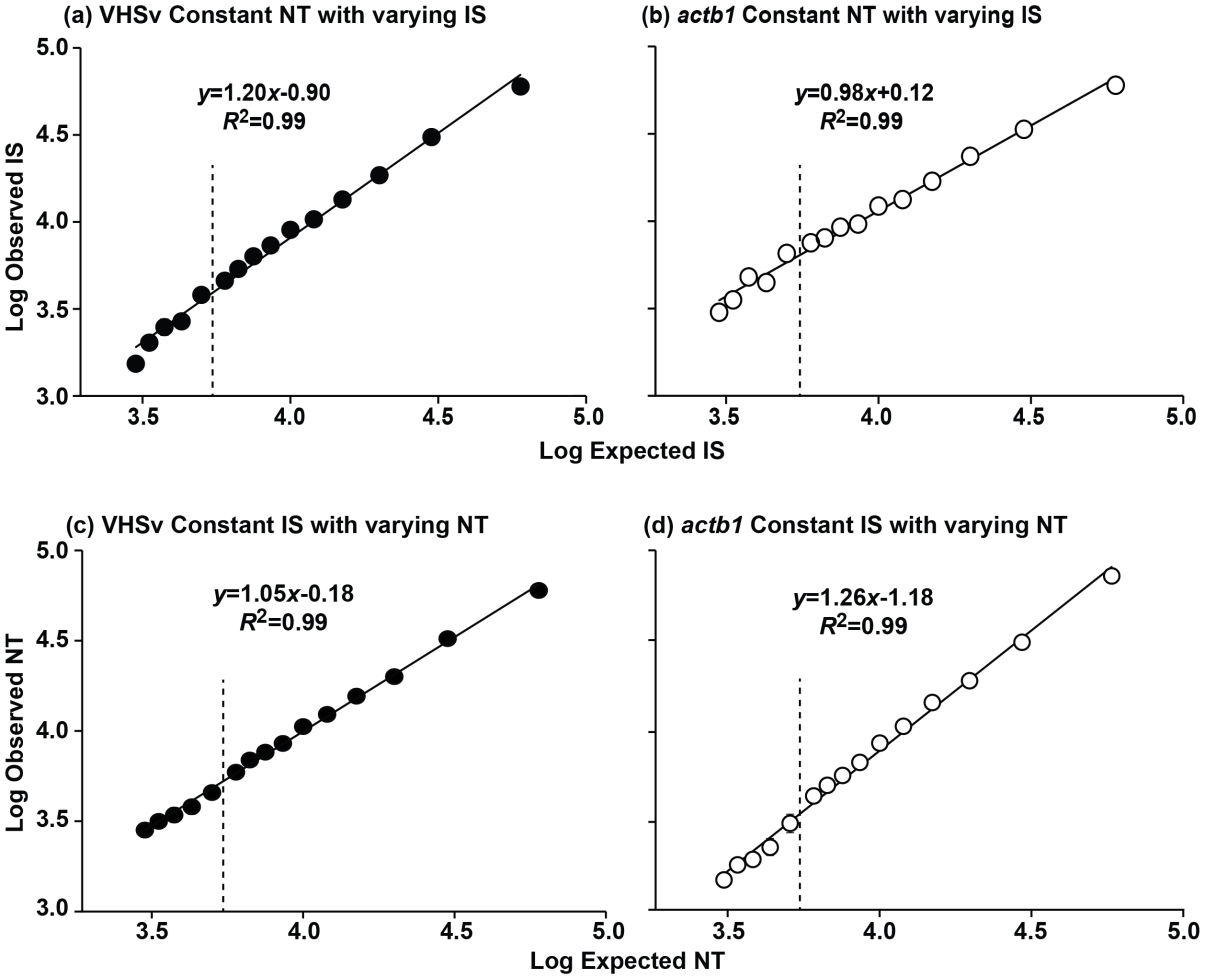
Relationship between the number of observed and expected molecules for NT:IS concentrations of 1∶1–1∶20. The concentration of Native Template (NT) is held constant and the Internal Standard (IS) varied for dilutions of: 1∶1 (6×10^4^ molecules), 1∶2, 1∶3, 1∶4, 1∶5, 1∶6, 1∶7, 1∶8, 1∶9, 1∶10, 1∶12, 1∶14, 1∶16, 1∶18, and 1∶20 (3×10^3^molecules). The 2-color fluorometric assay yields a linear relationship for (a) VHSv (*R* ^2^ = 0.99, *F = *1514.00, df = 1, 13, *p*<0.001) with a mean CV of 5% for dilutions 1∶1–1∶10 and 7% for concentrations down to 1∶20, and for (b) *actb1* (*R* ^2^ = 0.99, *F = *1283.00, df = 1, 13, *p*<0.001), CV = 5% and 7%. The same linear pattern is observed when the IS was held constant and NT varied for (c) VHSv (*R* ^2^ = 0.99, *F = *5124.00, df = 1, 13, *p*<0.001), CV = 5% for 1∶1–1∶10 and 7% for dilutions down to 1∶20, and (d) *actb1* (*R* ^2^ = 0.99, *F = *2434.00, df = 1, 13, *p*<0.001), CV = 3% and 6%. Error bars = standard error of results for triplicate runs. Dotted line = partition of dilutions from 1∶1–1∶10 (right) and 1∶12–1∶20 (left).

The relationship between the amount of PCR product remains linear when the concentration of NT is held constant and the IS is varied for both the VHSv *N*-gene (Figure 3a: *R* ^2^ = 0.99, *F = *1363.00, df = 1, 13, *p*<0.001) and the *actb1* gene (Figure 3b: *R* ^2^ = 0.99, *F = *1283.00, df = 1, 13, *p*<0.001). Figure 1b depicts the results that illustrate this relationship. The mean calculated CV is 5% for the VHSv *N*-gene over an NT:IS dilution range of 1∶1–1∶10 (yielding 6.0×10^4^±0.0×10^0^ to 4.6×10^3^±2.8×10^2^ molecules). The CV likewise is 5% for the *actb1* gene (yielding 6.0×10^4^±0.00×10^0^ to 7.5×10^3^±4.3×10^2^ molecules). At dilutions beyond 1∶10, the CV increases to 7% for both the VHSv *N*-gene (yielding up to 1.5×10^3^±1.1×10^2^ molecules) and the *actb1* gene (yielding up to 3.0×10^3^±1.2×10^2^ molecules) when the NT is held constant.

Analogous results are obtained when the IS is held constant and the NT is varied for the VHSv *N*-gene (Figures 1c and 3c: *R* ^2^ = 0.99, *F = *5124.00, df = 1, 13, *p*<0.001) and the *actb1* gene (Figure 3d: *R* ^2^ = 0.99, *F = *2434.00, df = 1, 13, *p*<0.001). The mean CV for the IS:NT dilution range of 1∶1–1∶10 is 5% for the VHSv *N*-gene (yielding 6.0×10^4^±0.0×10^0^ to 5.9×10^3^±2.2×10^3^ molecules) and 3% for the *actb1* gene (yielding 6.0×10^4^±0.0×10^0^ to 4.0×10^3^±4.3×10^1^ molecules). At dilutions beyond 1∶10, the CV increases to 7% for the VHSv *N*-gene (yielding up to 2.8×10^3^±1.8×10^2^ molecules) and 6% for the *actb1* gene (yielding up to 1.4×10^3^±6.2×10^1^ molecules) when the IS is held constant. Based on these findings, our quantifications are conducted in the range of 1∶10 to 10∶1 NT:IS to maximize accuracy.

**Figure 4:**
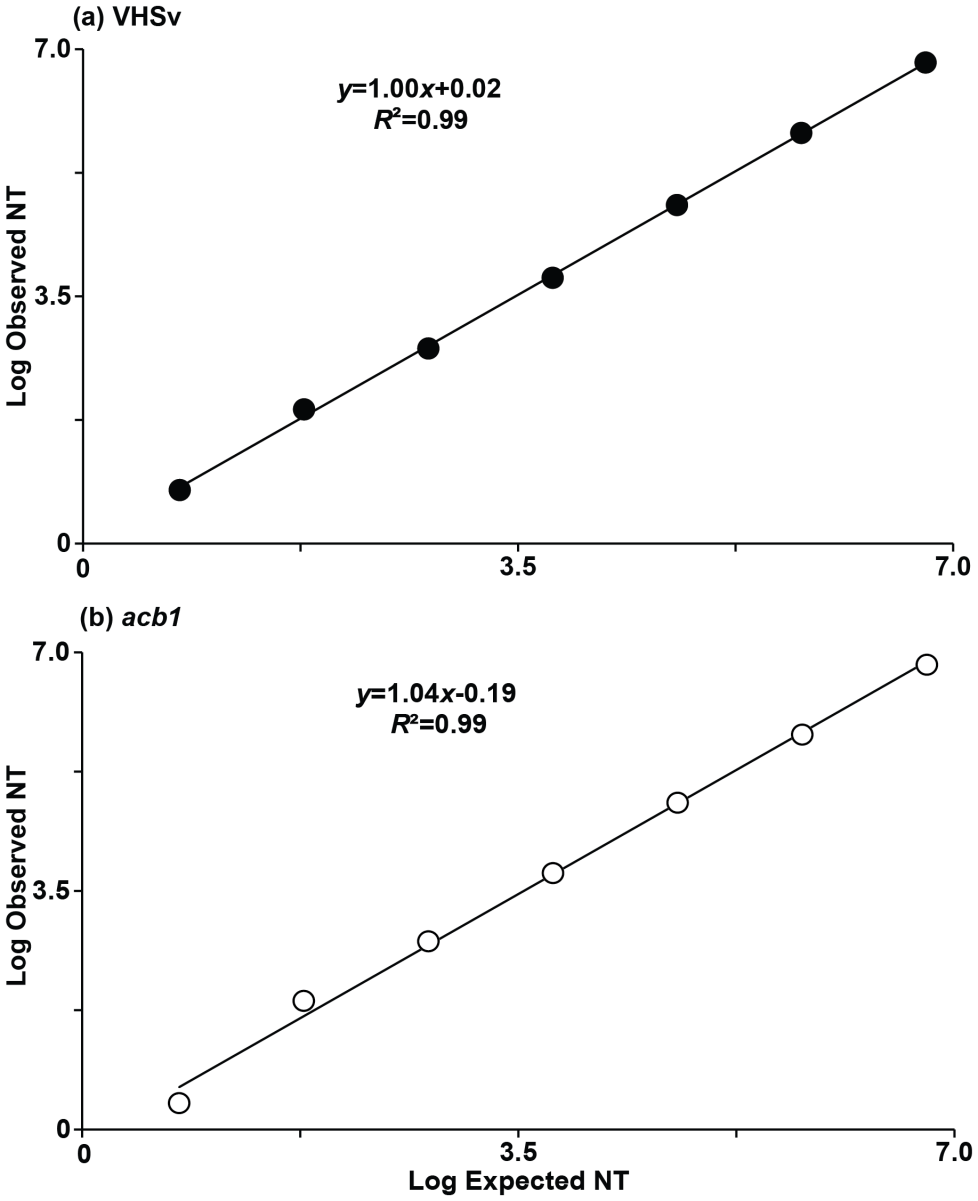
Relationship between the numbers of observed versus expected molecules when NT:IS concentrations are 1∶1. Results are based on dilutions of the Native Template (NT) and Internal Standard (IS) of 6×10^6^, 6×10^5^, 6×10^4^, 6×10^3^, 6×10^2^, 60, 6, and 0.6 molecules for VHSv and *actb1*. The 2-color fluorometric assay yields a linear relationship for (a) VHSv over seven orders of magnitude (from 6×10^6^ to 6×10^0^ VHSv molecules) with a slope of 1.00 (*R* ^2^ = 0.99, *F = *9404.00, df = 1, 5, *p*<0.001), and mean CV of 9%. A linear trend also is obtained for (b) *actb1* (*R* ^2^ = 0.99, *F = *1347.00, df = 1, 5, *p*<0.001). Slope = 1.04, mean CV = 10%. Error bars = standard error of triplicate runs.

Numbers of VHSv molecules show a linear relationship over seven orders of magnitude (serial dilutions of 6×10^6^ to 6×10^0^ molecules) when the NT:IS is 1∶1 (Figure 4a), with a slope of 1.00 (*R* ^2^ = 0.99, *F = *9404.00, df = 1, 5, *p*<0.001). Figure 1d illustrates this relationship, in which NT and IS increase by ≤3.2 C_t_ for each 10-fold serial dilution of the ESM. The mean CV for VHSv is estimated at 7% for samples of 6×10^6^ to 6×10^1^ molecules (measured as 6.5×10^6^±5.2×10^5^ to 7.9×10^1^±2.0×10^0^ molecules), and 9% when the range is extended to 6×10^0^ molecules (measured as 6.0×10^0^±1.0×10^0^ molecules). Results for *actb1* have a similar trend (Figure 4b), with a slope of 1.04 (*R* ^2^ = 0.99, *F = *1347.00, df = 1, 5, *p*<0.001), a mean CV of 7% for 6×10^6^ to 6×10^1^ molecules (measured as 6.6×10^6^±2.1×10^5^ to 7.8×10^1^±8.0×10^0^ molecules), and 10% when the range is extended to 6×10^0^ molecules (measured as 3.0×10^0^±0.4×10^0^ molecules). Stochastic sampling likely contributes to increased CV and S.E. in the measurements for 6 molecules.

### VHSv Detection and Quantification Comparison among Methods

**Figure 5:**
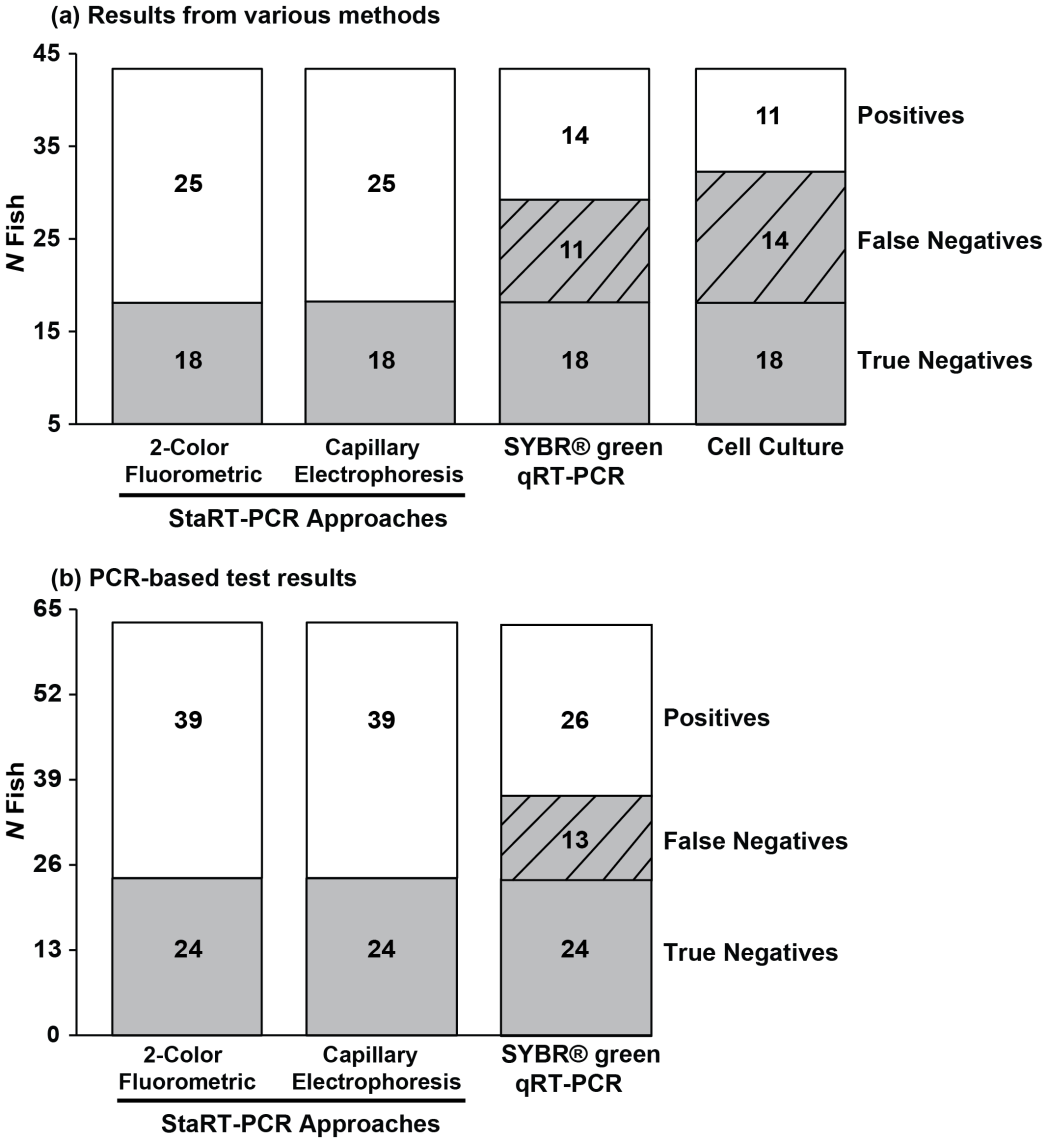
Relative numbers of VHSv positives and negatives from our 2-color fluorometric and capillary electrophoresis StaRT-PCR assays, which indicates identical numbers of positives and negatives. Compared to these tests, for 43 fishes (25 positives, 18 negatives (including 5 laboratory controls)), (a) SYBR® green has 44% false negative error (*χ* ^2^ = 5.67, df = 1, *p* = 0.02), and cell culture has 56% error (*χ* ^2^ = 9.36, df = 1, *p* = 0.002). For 63 fish samples (39 positives, 24 negatives (including 11 laboratory controls)), (b) SYBR® green qRT-PCR has 33% false negative error (*χ* ^2^ = 5.37, df = 1, *p* = 0.02), whereas the 2-color fluorometric and capillary electrophoresis tests show zero false negatives.

Results reveal that our present 2-color fluorometric real-time PCR assay and previous results from the Agilent capillary electrophoresis-based approach [37] both discriminate identical positives and negatives (i.e. they have the same accuracy; Figure 5; *χ* ^2^ = 0.00, df = 1, NS), and are free of false negatives (Figure 5). In contrast, the cell culture results have 56% false negative error (Figure 5a: *χ* ^2^ = 9.36, df = 1, *p* = 0.002) and SYBR® green yields 33–44% false negative error (Figure 5a,b: *χ* ^2^ = 5.37–5.67, df = 1, *p* = 0.02). All positives detected by SYBR® green qRT-PCR and cell culture also are positive with both of our StaRT-PCR methods (2-color fluorometric real time and capillary electrophoresis). The false negative range for SYBR® green qRT-PCR is 1.0×10^0^–1.6×10^2^ VHSv/10^6^ *actb1* molecules ( = 0.6×10^0^–2.5×10^2^ VHSv molecules, as quantified by our 2-color fluorometric method) and 1.0×10^0^–2.2×10^3^ VHSv/10^6^ *actb1* molecules ( = 0.6×10^0^–6.1×10^3^ VHSv molecules, as quantified by our 2-color fluorometric method) for cell culture. True negatives (including experimental controls) are negative with all assays; i.e. we find no false positives and no contamination.

**Figure 6:**
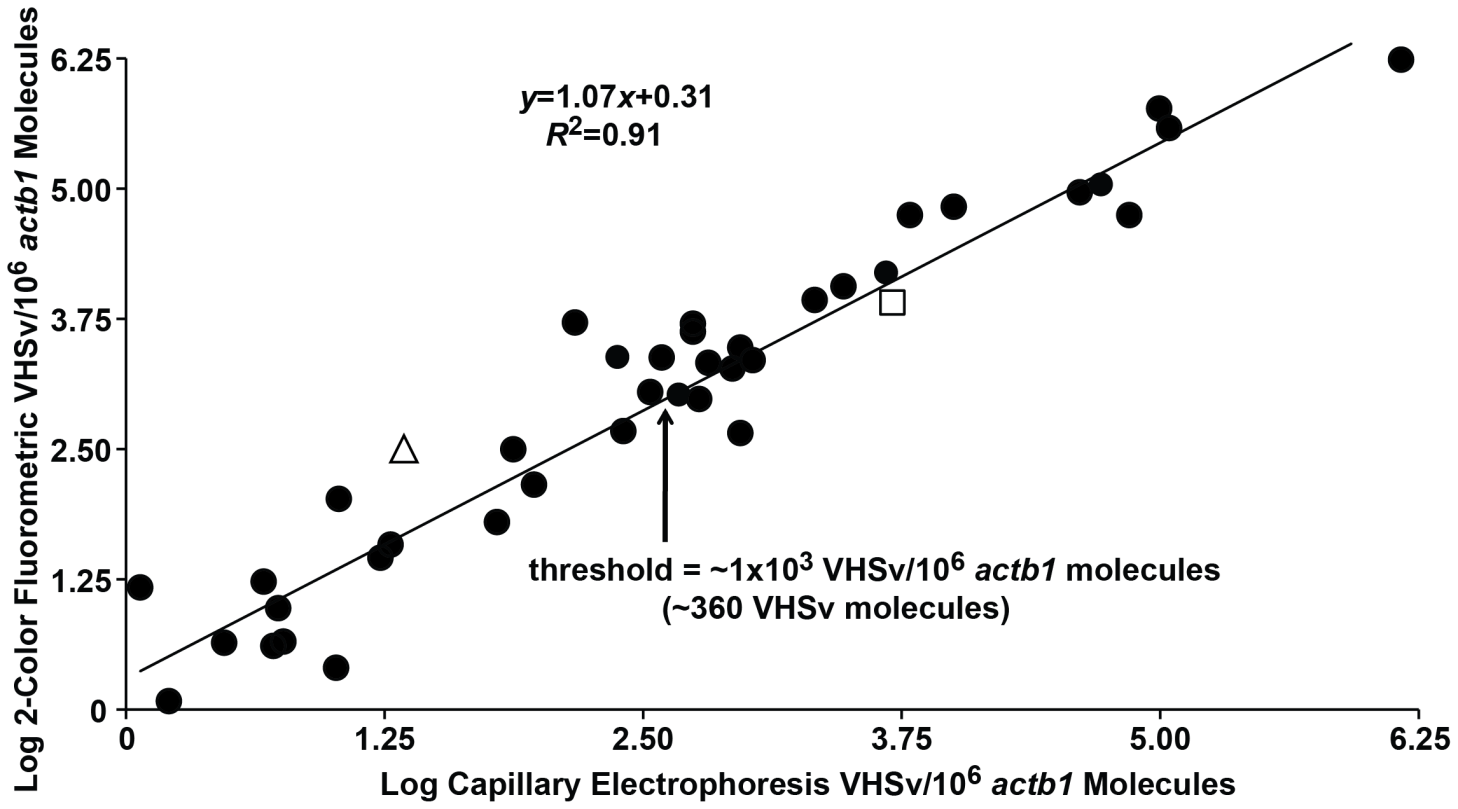
Mean log numbers of VHSv molecules/10^6^ *actb1* molecules from our new 2-color fluorometric assay versus the prior Agilent capillary electrophoresis approach. Results indicate a linear relationship between the two tests (*R* ^2^ = 0.91, df = 1, 38, *F = *396.40, *p*<0.001) and do not significantly differ (*t = *1.42, df = 78, NS). Arrow = Estimated threshold concentration of VHSv for fish with clinical signs of infection using our new assay, from a *χ* ^2^ test of nine symptomatic fish (1×10^3^ VHSv molecules/10^6^ *actb1* molecules = 3.6×10^2^ VHSv molecules). Triangle = false negative range for SYBR® green qRT-PCR (1.0×10^0^–1.6×10^2^ VHSv/10^6^ *actb1* molecules = 0.6×10^0^–2.5×10^2^ VHSv molecules). Square = false negative range for cell culture (1.0×10^0^–2.2×10^3^ VHSv/10^6^ *actb1* molecules = 0.6×10^0^–6.1×10^3^ VHSv molecules.

Numbers of VHSv molecules/10^6^ *actb1* molecules measured from the spleen tissues of positive fish are higher in the new assay, ranging to 1.21×10^6^ VHSv molecules/10^6^ *actb1* ( = 1.90×10^4^ VHSv molecules) than for the Agilent capillary-based test, which range to 8.4×10^5^ VHSv molecules/10^6^ *actb1* ( = 2.7×10^3^ VHSv molecules). However, both sets of values have a direct linear relationship (Figure 6: *R* ^2^ = 0.91, df = 1, 38, *F = *396.40, *p*<0.001, *t = *1.42, df = 78, NS). Muskellunge exhibiting clinical signs of infection contain a greater mean number of viral molecules (1.4×10^5^±6.5×10^3^ VHSv/10^6^ *actb1* molecules = 6.9×10^4^±6.9×10^3^ VHSv molecules) than those without (1.2×10^4^±1.7×10^3^ VHSv/10^6^ *actb1* molecules = 1.5×10^3^±1.6×10^2^ VHSv molecules). The estimated threshold at which those individuals display clinical signs of infection is ∼ 1×10^3^ VHSv/10^6^ *actb1* molecules ( = 3.6×10^2^ VHSv molecules) using our assay. Our sample sizes are not sufficient to further evaluate the relationship between this threshold number of molecules and clinical diagnosis (*χ* ^2^ = 0.09, df = 1, NS). Power analysis estimates that 52 fish samples (26 with and 26 without clinical signs) would be needed to verify this finding.

All data and analyses are publically accessible on the University of Toledo Lake Erie Center’s VHSv webpage (http://www.utoledo.edu/nsm/lec/research/glgl/VHS/VHS_main.html).

## Discussion

Disease diagnostic laboratories depend on rapid, sensitive, and accurate detection methods, which are easy to employ and cost-effective. Cell culture is the VHSv diagnostic approved by the World Organization of Animal Health [24], which takes up to a month to perform in clinical settings and often results in substantial false negatives – as revealed here and by other studies [26]–[28]. Compared with traditional cell culture, all PCR-based assays [32] – including the present one – show enhanced ability to detect VHSv since they amplify both the negative-strand non-replicating genomic RNA and the positive-strand replicating mRNA transcripts. Amplification of both transcripts may be beneficial since positive results may denote new spread of VHSv or latent cases in the geographic region where the samples are taken. This can aid in diagnosis of viral infections.

The present assay detects and quantifies VHSv-IVb in fishes from the Great Lakes using primers and probes that are homologous to the *N*-gene sequence of the widespread IVb isolate MI03GL and matches conserved sequence regions among all VHSv strains and substrains. Results demonstrate cross-reaction with all other VHSv strains and substrains tested. Other human and fish viruses do not amplify. Thus, our assay is VHSv-specific and detects all of its known variants.

Other PCR tests developed for VHSv by Chico et al. [26], López-Vázquez et al. [27], Liu et al. [29], Matejusova et al. [30], Cutrín et al. [31], Hope et al. [32], Garver et al. [33], and Jonstrup et al. [28], culminated in high numbers of false negatives, analogous to the SYBR® green test evaluated here (33–44% false negatives). Notably, 15–90% false negatives were reported by López-Vázquez et al. [27], 25–92% by Chico et al. [26], and values to 42% by Jonstrup et al. [28] for their respective approaches. Unlike those other real-time PCR tests for VHSv [26]–[34], our method incorporates intrinsic quality control standards (IS) to circumvent false negative results.

Specifically, exogenous (IS) and endogenous controls (the commonly used reference gene *actb1*) facilitate optimal detection of true positives and act to normalize the quantification of viral molecules. Use of IS is recommended by the International Organization for Standardization [46], the U.S. Environmental Protection Agency [47], and the U.S. Food and Drug Administration [48]. Tests for Hepatitis C virus [49] and Human Immunodeficiency virus [50], [51] already have implemented IS in their assays.

Our assay is sensitive, follows a linear relationship with increasing viral concentration, and is highly reproducible. It detects down to five VHSv *N*-gene molecules with 100% accuracy, based on Poisson distribution. Other real-time PCR assays for VHSv had much higher detection thresholds. Notably, Liu et al.'s [29] test required ≥140 viral copies of VHSv, and assays by Hope et al. [32] and Garver et al. [33] needed ≥100 viral copies. Our results are consistent for samples containing six to 6,000,000 VHSv molecules. Stochastic variation is evident only at extremely low dilutions (< five molecules). Results confirm reliability from concentrations of 1∶1 to 1∶20 NT:IS, with some slight increase in CV at dilutions >1∶10. We thus recommend adjusting the relative concentrations of NT:IS to maximize accuracy, following recommendations in the Materials and Methods section “Performing the VHSv Assay”. All quantification values reported here fall within this 1∶10 range, which allows us to distinguish ∼ a 1.25-fold C_t_ difference. Our assay also should work well with highly degraded samples (e.g. dead fish in the field), as described for human cancer samples using this type of approach by some of our team members [unpublished data].

This 2-color fluorometric real-time assay is highly accurate and free of the size separation steps required for our previously-published Agilent capillary electrophoresis approach [37]. Here we determine higher numbers of VHSv molecules for the same fish samples, due to the re-design of primers and use of fluorescent-labeled probes. Results from both methods have a linear relationship and are readily cross-calibrated.

Laboratory challenged muskellunge showing clinical signs of infection have a greater mean number of viral molecules than those without. It is estimated that ∼ 1×10^3^ VHSv/10^6^ *actb1* molecules ( = 3.6×10^2^ VHSv molecules) appears to mark a clinical threshold for signs of VHSv. However, exhibition of clinical signs at this biomarker could be species-specific, and may differ between fish in the laboratory and those in the field. Further experiments are warranted to validate this assumption.

### Conclusions

Our assay is highly sensitive and accurate, free of false negatives, and reliably quantifies a wide range of VHSv in fish tissue samples. Other PCR-based methods and cell culture techniques had high proportions of false negatives since they lacked intrinsic quality control, which could lead to spread of the virus. This new test will aid rapid, accurate, and low-cost diagnosis of the disease. It has wide applicability across the geographic range of the virus, and should be highly successful in elucidating new occurrences and circumventing spread.
